# Regeneration of Subcutaneous Cartilage in a Swine Model Using Autologous Auricular Chondrocytes and Electrospun Nanofiber Membranes Under Conditions of Varying Gelatin/PCL Ratios

**DOI:** 10.3389/fbioe.2021.752677

**Published:** 2021-12-21

**Authors:** Rui Zheng, Xiaoyun Wang, Jixin Xue, Lin Yao, Gaoyang Wu, Bingcheng Yi, Mengjie Hou, Hui Xu, Ruhong Zhang, Jie Chen, Zhengyu Shen, Yu Liu, Guangdong Zhou

**Affiliations:** ^1^ Department of Plastic and Reconstructive Surgery, Shanghai 9th People’s Hospital, Shanghai Jiao Tong University School of Medicine, Shanghai Key Laboratory of Tissue Engineering, Shanghai Stem Cell Institute, Shanghai, China; ^2^ Department of Dermatology, Shanghai 9th People’s Hospital, Shanghai Jiao Tong University School of Medicine, Shanghai, China; ^3^ Department of Cosmetic Surgery, Tongren Hospital, Shanghai Jiao Tong University School of Medicine, Shanghai, China; ^4^ Department of Orthopaedics, The Second Affiliated Hospital and Yuying Children’s Hospital of Wenzhou Medical University, Wenzhou, China; ^5^ National Tissue Engineering Center of China, Shanghai, China; ^6^ Research Institute of Plastic Surgery, Weifang Medical College, Weifang, China

**Keywords:** cartilage regeneration, large animal, subcutaneous environment, electrospun nanofiber membrane, inflammatory reaction

## Abstract

The scarcity of ideal biocompatible scaffolds makes the regeneration of cartilage in the subcutaneous environment of large animals difficult. We have previously reported the successful regeneration of good-quality cartilage in a nude mouse model using the electrospun gelatin/polycaprolactone (GT/PCL) nanofiber membranes. The GT/PCL ratios were varied to generate different sets of membranes to conduct the experiments. However, it is unknown whether these GT/PCL membranes can support the process of cartilage regeneration in an immunocompetent large animal model. We seeded swine auricular chondrocytes onto different GT/PCL nanofiber membranes (GT:PCL = 30:70, 50:50, and 70:30) under the sandwich cell-seeding mode. Prior to subcutaneously implanting the samples into an autologous host, they were cultured *in vitro* over a period of 2 weeks. The results revealed that the nanofiber membranes with different GT/PCL ratios could support the process of subcutaneous cartilage regeneration in an autologous swine model. The maximum extent of homogeneity in the cartilage tissues was achieved when the G5P5 (GT: PC = 50: 50) group was used for the regeneration of cartilage. The formed homogeneous cartilage tissues were characterized by the maximum cartilage formation ratio. The extents of the ingrowth of the fibrous tissues realized and the extents of infiltration of inflammatory cells achieved were found to be the minimum in this case. Quantitative analyses were conducted to determine the wet weight, cartilage-specific extracellular matrix content, and Young’s modulus. The results indicated that the optimal extent of cartilage formation was observed in the G5P5 group. These results indicated that the GT/PCL nanofiber membranes could serve as a potential scaffold for supporting subcutaneous cartilage regeneration under clinical settings. An optimum GT/PCL ratio can promote cartilage formation.

## 1 Introduction

Cartilage defects in the subcutaneous environment (such as ear, nose, and trachea) due to congenital disease (microtia), cancer removal, or trauma are very common in plastic and reconstructive surgery (Wiggenhauser et al., 2017). The defects can significantly alter the appearance, psychological status, and physiological function of the patients. Traditional methods followed to repair these defects involve the implantation of a pre-shaped prosthetic graft made from silastic or high-density polyethylene (Medpor^®^) or the transplantation of sculpted autologous cartilage (usually harvested from the rib, nasal septum, or auricle) (Brent, 1999; Bateman and Jones, 2000; Kim, et al., 2011). The poor bioactivity of the prosthetic grafts can potentially result in extrusion and infection, while the use of grafts developed from autologous cartilage can cause severe donor-site morbidity. The process is characterized by a prolonged operative time, and the success of the process is heavily dependent on the skill of the surgeon. An ideal cartilaginous substitute with a pre-designed shape and characterized by the minimum donor-site morbidity can be developed using tissue engineering technology. This can help address the problems faced when the traditional cartilage reconstructive approaches are followed (Sterodimas et al., 2009; Bichara et al., 2012). Recently, good progress has been made in the field of cartilage engineering. Materials and methods that can be used for nasal, auricular, and tracheal reconstruction have been developed. Some of the materials developed have entered the stage of proof-of-feasibility clinical trials (Zang et al., 2013; Fulco et al., 2014; Bichara et al., 2014; Pomerantseva et al., 2016). However, a method to achieve reliable and stable cartilage regeneration in the subcutaneous environment is yet to be developed. This is because the clinical outcomes vary from patient to patient. The fluctuations in the clinical outcomes can be potentially attributed to the inflammatory reactions triggered by the use of the engineered cartilaginous grafts for subcutaneous implantation.

The subcutaneous environment is different from the environment of the immune privileged articular tissue (Huang et al., 2016). The subcutaneous environment is characterized by a high extent of immune activities and the absence of endogenous chondrogenic cues (Nayyer et al., 2012). The implanted engineered cartilaginous grafts face acute immune attack, which may significantly hinder the process of cartilage formation, resulting in the absorption of the implanted grafts. This phenomenon is particularly observed when the grafts engineered from polymeric scaffolds such as polyglycolic acid/polylactic acid (PGA/PLA), whose degradation products promote antigenicity, are used (Asawa et al., 2012). We have previously proposed a long (>8 weeks) *in vitro* pre-culture scheme that can be used to implant the engineered grafts when the scaffolds have been significantly degraded. The extent of postimplantation inflammation realized could be decreased, and stable subcutaneous cartilage could be formed in an autologous preclinical goat model using the PGA/PLA-engineered cartilage (Liu et al., 2016). However, the process of prolonged *in vitro* culture results in an increase in the grafting time. This can potentially increase the risk of contamination. Under these conditions, the cost of executing the method also increases. It is important to identify or develop a scaffold characterized by low immunogenicity to support the formation of stable subcutaneous cartilage and reduce the time taken for the *in vitro* pre-culture process.

Electrospun nanofibers can mimic microscopic aspects of the extracellular matrix (ECM) and help to establish a tissue-specific microenvironment that maintains and regulates cell behavior and function (Kanani and Bahrami, 2010; Qian et al., 2018). Gelatin (GT) and polycaprolactone (PCL) exhibit significantly low immunogenicity. GT is a biosafety scaffold that exhibits good biocompatibility but poor mechanical strength. The scaffold is characterized by a rapid degradation time. PCL is a synthetic polymer characterized by a good mechanical strength and prolonged degradation time. It can be used to complement the disadvantages of using GT. Electrospun nanofibers fabricated by blending GT with PCL exhibit excellent physiochemical, biomechanical, and biocompatible properties. These nanofibers have been widely used as scaffolds to conduct *in vitro* cellular studies with a variety of tissues (Chong et al., 2007; Ji et al., 2013; Jiang et al., 2017). There are a few papers that report the feasibility of engineering a three-dimensional (3D) tissue using the electrospun GT/PCL membranes. The lack of information can be potentially attributed to the limitations posed by the thicknesses of the materials. There is also a dearth of information in the field of 3D tissue regeneration in large animals used in preclinical trials. We have previously reported the ability of the electrospun GT/PCL membranes to support cartilage formation following a sandwich strategy using nude mouse models (Xue et al., 2013; Zheng et al., 2014). The results revealed that a combination of GT and PCL (high GT and low PCL contents; aGT: PCL = 70:30) could be used to achieve cartilage formation in nude mice. Successful regeneration of human ear-shaped cartilage could be achieved (Zheng, et al., 2014).

However, it is unknown if the formation of cartilage can be realized using this scaffold in a large animal model used in preclinical trials. It should also be tested if the optimized GT/PCL ratio used for cartilage regeneration in a nude mouse can be effectively used for cartilage regeneration in a preclinical large animal model. It has been reported that the electrospun nanofibrous membranes function as anti-inflammatory barriers that help alleviate inflammatory reactions (Wang et al., 2016; Luo et al., 2016). We also studied if the electrospun GT/PCL membranes can be used to alleviate inflammatory reactions to improve the quality of the 3D cartilage regenerated in a large animal model.

We used the electrospun GT/PCL membranes with different GT/PCL ratios to construct cartilage-like tissues (*in vitro*) using the previously established sandwich strategy to predict the potential of using the scaffold in the field of clinical translation. Following this, the regenerated cartilage-like tissue was implanted into an autologous subcutaneous environment in a swine model to evaluate the feasibility of forming subcutaneous cartilage and study the effect of different GT/PCL ratios on the cartilage regeneration process in a large preclinical model. The results presented herein provide direct experimental evidence required to promote the clinical translation of cartilage regeneration using electrospun GT/PCL membranes.

## 2 Materials and Methods

### 2.1 Isolation and Expansion of Chondrocytes

A total of five adult swine (16 weeks old; Shanghai Jiagan Biological Technology Co., Ltd., Shanghai, China) were used for the studies. All animals received humane care according to the guidelines laid down in 2006 by the National Ministry of Science in the “Guide for Care of Laboratory Animals.” The animal care and experiment committee of the Shanghai Jiao Tong University School of Medicine approved the animal studies conducted by us. A biopsy of the auricular cartilage (2.0 × 1.0 cm) obtained from the autologous swine sample was conducted under conditions of endotracheal anesthesia. The surrounding fibrous tissue and perichondrium were removed, and the cartilage biopsy was minced into fragments (dimension: 1.0 mm^3^). The samples were pretreated with 0.25% trypsin (HyClone, Logan, UT, USA) at 37°C over a period of 30 min. Following this, the sample was washed with phosphate-buffered solution (PBS). Subsequently, the sample was digested with 0.3% collagenase NB4 (Worthington Biochemical Corp., Freehold, NJ, United States) for 8 h at 37°C. The isolated cells were cultured and expanded using Dulbecco’s modified Eagle’s medium (DMEM, Gibco BRL, Grand Island, NY, United States) containing 10% fetal bovine serum (FBS, HyClone, Logan, UT, United States), penicillin (100 U/ml), and streptomycin (100 μg/ml), following previously reported protocols (Feng et al., 2012). Chondrocytes in passage two (P2) were harvested for scaffold seeding.

### 2.2 Preparation of Scaffolds Under Conditions of Varying GT/PCL Ratios

As previously described (Xue et al., 2013; Zheng et al., 2014), GT/PCL membranes with GT:PCL ratios (by weight) of 70:30 (G7P3), 50:50 (G5P5), and 30:70 (G3P7) were fabricated and trimmed to form round-shaped samples (diameter: 9 mm). The samples were irradiated with UV light over a period of 30 min to obtain sterilized samples. Prior to conducting the cell seeding process, the samples were lyophilized using a vacuum free-drier (VirTis BenchTop 6.6; SP Industries, Gardiner, NY, United States).

### 2.3 Mechanical Analysis of the Scaffolds

The mechanical properties of the electrospun fibrous membranes in their dry state were determined using a tabletop uniaxial material testing machine (H5K-S, Hounsfield, United Kingdom). Rectangular specimens (10 × 50 mm) were stretched at a constant cross-head speed of 10 mm/min (Feng et al., 2012). At least eight samples were tested for each type of membrane. The tensile strength, Young’s modulus, and strain at break for all the groups were calculated and analyzed based on the stress–strain curve.

### 2.4 Scanning Electron Microscopy

For the cell-loaded group, 2.0 × 10^5^ chondrocytes in 1.0 ml of the medium (DMEM with 10% FBS) were evenly dropped onto the GT/PCL membrane and incubated at 37°C under conditions of 95% humidity and 5% CO_2_ (incubation time: 24 h). The cell-loaded or non-loaded membranes were rinsed with PBS, fixed in 0.05% glutaraldehyde at 4°C overnight, dehydrated through a graded series of ethanol, critical-point dried, and analyzed using the SEM technique (JEOL-6380LV, Tokyo, Japan). The extent of adhesion achieved and the distribution of the chondrocytes on the membranes were studied following previously reported methods (Xue et al., 2013; Zheng et al., 2014).

### 2.5 Macrophages and GT/PCL Membranes

#### 2.5.1 Macrophage Seeding

GT/PCL membranes were punched into 14-mm discs to fit 24-well plates. The samples were disinfected with 70% ethanol and washed thoroughly with PBS prior to conducting the cell seeding process. Raw 264.7 macrophages at 80% confluence were detached using trypsin. Subsequently, the cells were centrifuged and resuspended in the medium. The seeding density of the macrophage on the scaffolds was 5 × 10^5^ cells/cm^2^. The cell-seeded scaffolds were transferred to an incubator and cultured at 37°C. The medium was changed every 2 days. The samples were harvested at predesignated time points for further analysis.

#### 2.5.2 Confocal Microscopy

The biocompatibility of the macrophages with the GT/PCL membranes was analyzed using the confocal microscope (Leica, TCS SP8 STED 3X, Wetzlar, Germany) technique. On day 2, the cell-seeded scaffolds were harvested and fixed with 4% paraformaldehyde and permeabilized with 0.1% Triton X-100 (time: 5 min). Subsequently, the treated samples were incubated with rhodamine-conjugated phalloidin for 30 min at room temperature in the dark. DAPI was used to stain the nuclei. The samples were imaged using a confocal microscope.

#### 2.5.3 Enzyme-Linked Immunosorbent Assay

The supernatant obtained when the macrophages were cultured was collected after 24 h of incubation. The supernatant was used to conduct ELISA. Mouse IL-6 ELISA Kit, Mouse TNF-α ELISA Kit, and Human/Mouse Arginase 1/ARG1 ELISA Kit (all ELISA kits: MultiSciences, Hangzhou, China) were used for the studies. The protocols outlined by the manufacturers were followed. The macrophages directly seeded inside the 14-mm dishes were used as the negative controls. The macrophages treated with a solution of lipopolysaccharide (LPS; 2.5 μg/ml) were used as the positive controls. Cell culture experiments were performed in groups of four, and the experiments were repeated three times (Zheng et al., 2021).

#### 2.5.4 Histological Analysis

Paraformaldehyde fixed macrophage-seeded GT/PCL membranes (14 mm diameter) were embedded in paraffin at days 2, 4, and 6. Sections were stained with hematoxylin and eosin (H&E) (Wang et al., 2021).

### 2.6 Preparation of Chondrocyte-Scaffold Constructs

The chondrocyte-GT/PCL constructs were prepared following the sandwich approach reported in the literature (Gong et al., 2011). Briefly, a chondrocyte suspension (5 μl) with a cell density of 100 × 10^6^ cells/ml was seeded onto one slice of the GT/PCL membrane. The process was followed by stacking another slice of the GT/PCL membrane onto the previous one. Subsequently, it was seeded with the same number of chondrocytes until a 10-layer membrane stack was formed. The constructs were incubated for 2.5 h (37°C, 95% humidity, 5% CO_2_) to allow the cells to get attached. Following this, a pre-warmed medium (DMEM with 10% FBS) was applied gently to cover the constructs. The medium was changed twice per week. After 2 weeks, the constructs were either subjected to the conditions of gross, histological, and immunohistochemical analyses or subcutaneously implanted into autologous swine members to conduct *in vivo* tests.

### 2.7 Subcutaneous Implantation

After 2 weeks of *in vitro* culture, the chondrocyte-GT/PCL constructs were implanted subcutaneously into autologous swine (time: 3 and 6 weeks). Under conditions of endotracheal anesthesia, individual subcutaneous pockets were created in the abdominal area. One construct was implanted into each pocket. The incisions were closed, and the animals were allowed to recover. Penicillin was injected intramuscularly over a period of 3 days, commencing on the first-day post operation.

### 2.8 Gross Evaluation

The *in vivo* samples were harvested after 3 and 6 weeks had passed post subcutaneous implantation. The surrounding fibrous tissues were carefully removed, and the appearance (color and texture) and wet weight of the samples were studied.

### 2.9 Histological and Immunohistochemical Analyses

All the constructs targeted for histological analysis were bisected post gross evaluation. One-half of each construct was stored at −80°C for biochemical assays. The other half was fixed with 4% paraformaldehyde (fixing time: 24 h), embedded in paraffin, sectioned into slices (diameter: 5 μm), and mounted on glass slides. Sections were stained with hematoxylin and eosin (H&E) or Safranin O following standard protocols. Type II collagen, CD3, and CD68 were detected following the principles of immunohistochemistry. Mouse anti-human type II collagen monoclonal antibody (1:200 in PBS, Santa Cruz, CA, United States), mouse anti-human CD3 monoclonal antibody (1:200 in PBS, Santa Cruz, CA, United States), and mouse anti-human CD68 monoclonal antibody (1:200 in PBS, Santa Cruz, CA, United States) were used to study all the samples. Horseradish peroxidase (HRP)-conjugated anti-mouse antibody (1:200 in PBS, Santa Cruz, CA, United States) was then applied as a secondary antibody. Color development was achieved using diaminobenzidine tetrahydrochloride (DAB, Santa Cruz, CA, United States), and cell nuclei were counterstained with hematoxylin according to previously established techniques (Liu et al., 2016).

### 2.10 Biomechanical and Biochemical Analyses

Young’s moduli of thatic compression; biomechanical analyzer, Instron-5542, Canton, MA, United States) (Yan, et e 3- and 6-weeks specimens were detected and analyzed following previously established methods (Yan, et al., 2009). After mechanical testing, the samples were collected and minced for biochemical analysis. The sulfated glycosaminoglycan (GAG) content was determined by conducting the dimethylmethylene blue chloride (DMMB, Sigma-Aldrich, St. Louis, MO, United States) assay, and the results were expressed in mg/g wet weight (Farndale et al., 1982). The content of type II collagen was determined following an enzyme-linked immunosorbent assay approach (Zhou et al., 2018). Five samples were analyzed in each group, and all assays were performed in duplicate as per the manufacturer’s instructions.

### 2.11 Semi-Quantification Analysis

The areas corresponding to cartilage formation, undegraded scaffold, and inflammatory infiltration were quantified using the software used for picture analysis (ImageJ, National Institutes of Health (NIH), Bethesda, MD, United States). The corresponding regions were studied by analyzing the specific histological features. Subsequently, the total area in each structure was summed, and the corresponding areas of each parameter were divided by the total area of the samples.

### 2.12 Statistical Analysis

The quantitative data were recorded as mean ± standard deviation (SD). The differences among the groups in wet weight, GAG content, type II collagen content, and Young’s moduli (for the 3- and 6-week specimens) were analyzed by conducting the one-way ANOVA tests (*n* = 5). The area proportions corresponding to the cartilage tissue, undegraded scaffold, and inflammatory infiltration recorded for the 3-week specimens were also analyzed by conducting the one-way ANOVA tests (*n* = 5). A *p*-value less than 0.05 was considered statistically significant.

## 3 Results

### 3.1 Influence of the GT/PCL Ratio on the Basic Properties of the Scaffolds and Chondrocyte Compatibility

**FIGURE 1:**
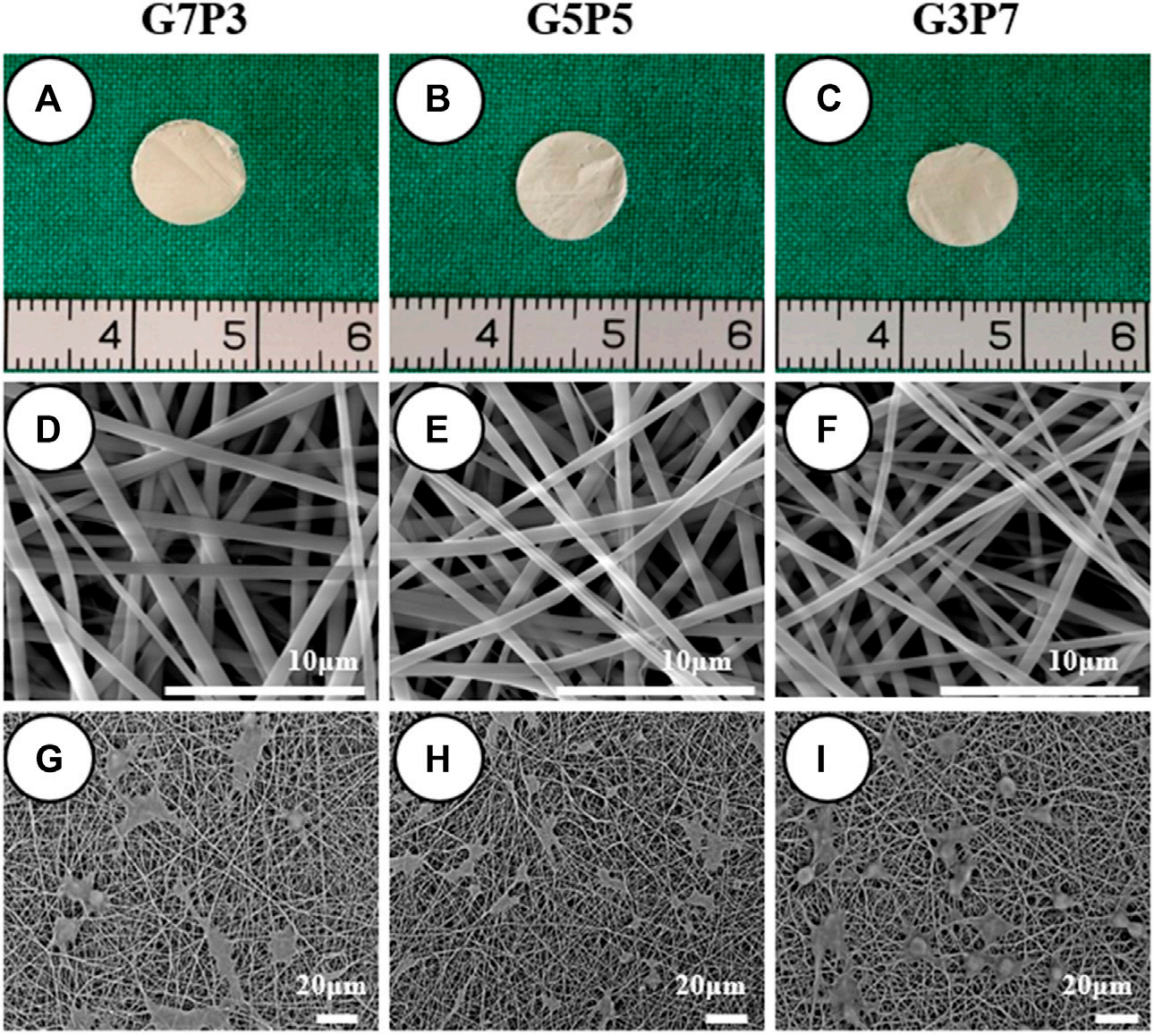
Gross view, microstructure, and cell compatibility of GT/PCL membranes. The membranes are easily trimmed into round shape, and no visible differences are observed among different GT/PCL content groups **(A–C)**. SEM shows a similar fibrous microstructure while the fiber diameter slightly decreased with GT content **(D–F),** and chondrocytes can spread well on the surfaces of all the membranes after 24 h of cell seeding **(G–I)**.

We first examined the gross appearance, microstructure, and chondrocyte compatibility of the GT/PCL membranes. The results revealed that the GT/PCL membranes characterized by varying GT/PCL ratios appeared ivory-white. The diameters of the membranes were calculated to be 9 mm (gross observation; Figures 1A–C). Analysis of the SEM images revealed that the diameter of the fiber decreased slightly with an increase in the GT content (Figures 1D–F). Additional mechanical analyses were conducted. Significant differences in the mechanical properties were observed among the three groups of the GT/PCL membranes. The tensile strengths and Young’s moduli of the membranes increased significantly while the strain at break decreased with an increase in the PCL content (Supplementary Figure S1). The chondrocytes adhered and spread well on the membranes in all groups after 24 h of cell seeding. This indicated that all the GT/PCL membranes exhibited good biocompatibility with the chondrocytes (Figures 1G–I).

### 3.2 Influence of the GT/PCL Ratio on the Biocompatibility, Immunomodulatory Behavior, and Cell Distribution of the Macrophages (*in vitro*)

Analysis of the confocal microscopy images revealed that the macrophages grew well on the membranes with different GT/PCL contents after 24 h of incubation (Supplementary Figures S2A–C). No significant differences in TNF-α (M1 marker), IL-6 (M1 marker), and Arg-1 (M2 marker) were observed among the three groups, indicating that the GT/PCL membranes characterized by varying GT/PCL ratios exhibited low immunogenicity. The results also revealed the difficulty in realizing the polarization of the macrophages (Supplementary Figures S2D–F). Results from histological analyses revealed that the macrophages grew well on the surface of the GT/PCL membranes. It was also observed that the macrophages could not infiltrate the membranes even after 2 and 4 days (Supplementary Figures S3A–F). This indicated that the membranes provided a physical barrier that blocked macrophage infiltration. When G5P5 and G3P7 were studied, it was observed that the effect of the cell barrier could be observed at day 6 (Supplementary Figures S3H,I). For the G7P3 group, a small number of the macrophages could be detected inside the membranes at day 6 (Supplementary Figure S3G). This indicated that the **barrier effect** started to deteriorate under these conditions. This could be attributed to the rapid degradation of the G7P3 membranes.

### 3.3 Influence of the GT/PCL Ratio on the Process of *in vitro* Cartilage Formation

**FIGURE 2:**
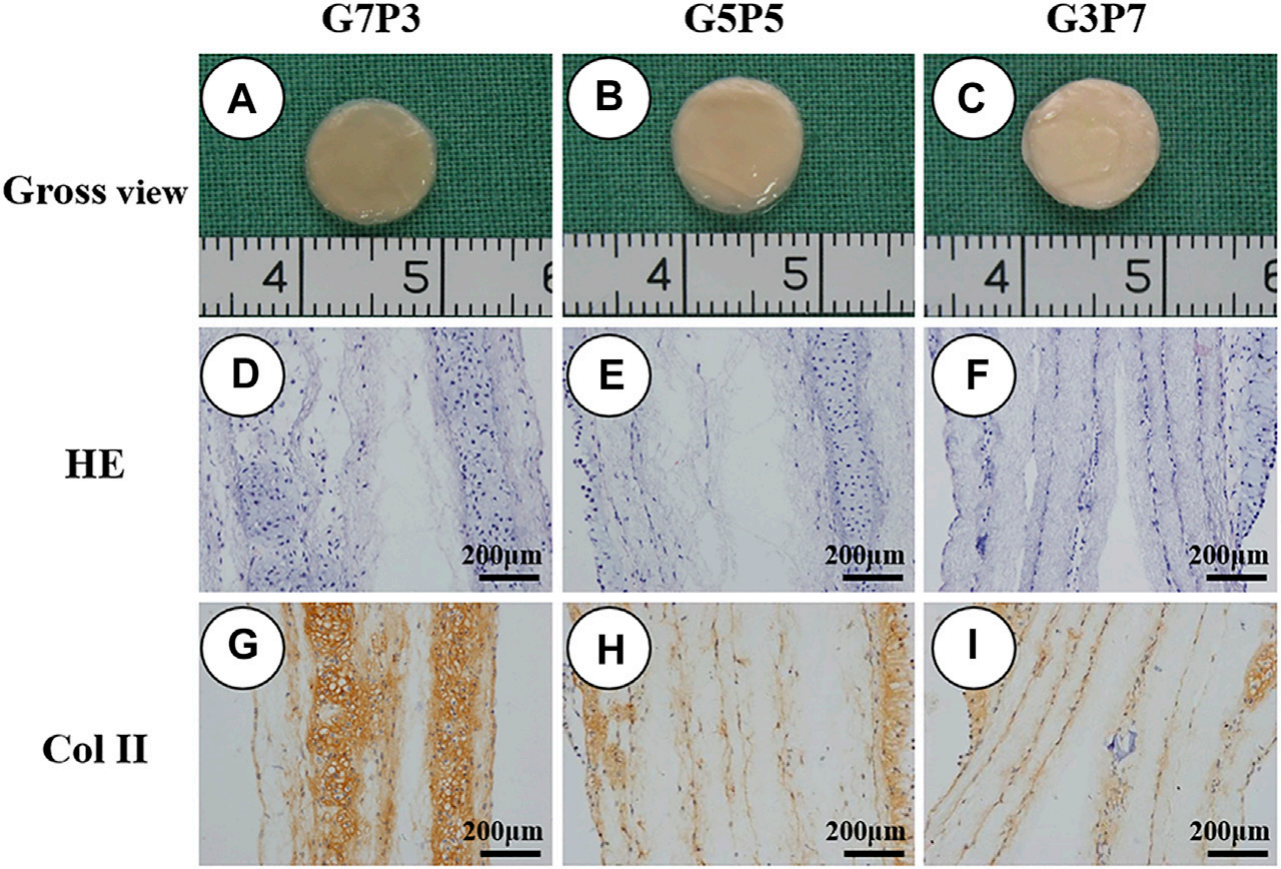
Gross view and histological examination of engineered tissues at 2 weeks *in vitro*. All the samples basically maintain the cylinder shape after cell seeding and retain the original shape after *in vitro* implantation **(A–C)**. The samples in all groups form cartilage-like tissue with typical lacuna structure and positive staining of type II collagen **(D–I)**. In the G7P3 group **(G)**, the samples show obviously positive staining of type II collagen than the other two groups **(H,I)**.

Before conducting the process of subcutaneous implantation, the samples generated *in vitro* were studied for cartilage formation following the processes of gross observation and histological staining. It was observed that the chondrocyte-GT/PCL constructs in all groups retained their original shapes and sizes when they were subjected to conditions of *in vitro* culture over 2 weeks (Figures 2A–C). Histologically, all the samples formed cartilage-like tissues with typical lacuna-like structures. The type II collagen could be positively stained (Figures 2D–I). The extent of ECM deposition observed in the cartilage present in the G7P3 group was higher than the extent of cartilage ECM depositions observed in the other two groups. This was revealed by analyzing the staining patterns of type II collagen (Figure 2G). In addition, the samples belonging to the G7P3 group exhibited better neocartilage integration. These samples did not exhibit the lamellar structure. Relatively loose lamellar structures separated by the GT/PCL membranes were observed in the G3P7 group. The results obtained from the *in vitro* studies indicated that the GT/PCL membranes characterized by low PCL contents could promote the formation of cartilage *in vitro*.

### 3.4 Influence of the GT/PCL Ratio on the Early Stage of Subcutaneous Cartilage Formation

**FIGURE 3:**
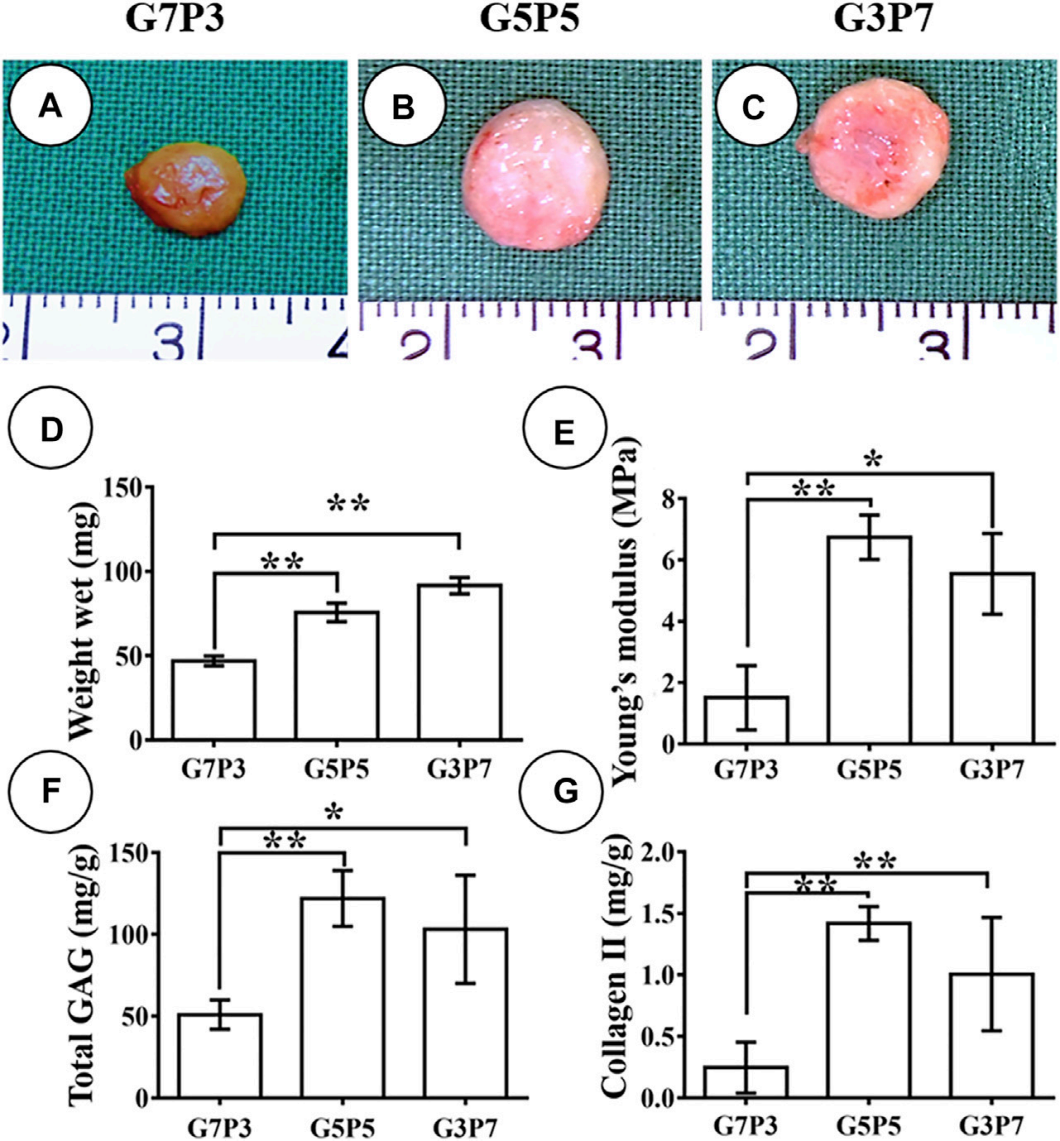
Gross view and quantitative analysis of engineered tissues at 3 weeks *in vivo*. The samples in G7P3 remarkably shrink and show yellowish appearance **(A)** while the samples in G5P5 and G3P7 groups retained the original size and show ivory-white appearance **(B,C)**. Quantitative analysis demonstrates that the samples in the G7P3 group show lower values in wet weight, Young’s modulus, total GAG, and Collagen Ⅱ than those in G5P5 and G3P7 groups **(D–G)**. No obvious differences are observed in terms of wet weight, Young’s modulus, total GAG, and Collagen Ⅱ between the G5P5 group and the G3P7 group **(D–G)**. *Indicating significant differences (*p < 0.05; **p < 0.01).

We studied the early stages of cartilage formation *in vivo* using the GT/PCL membranes under conditions of varying GT/PCL ratios. We used a swine model for our studies. After 3 weeks of subcutaneous implantation (in autologous swine), the samples belonging to the G7P3 group shrank significantly and appeared yellow. The texture of the samples was soft (Figure 3A). Under the same conditions, the samples belonging to the G5P5 and G3P7 groups retained the original sizes and formed cartilage-like tissues that were hard and elastic (Figures 3B,C). The wet weight, Young’s moduli, GAG content, and Collagen Ⅱ content recorded for the G7P3 group were lower than those recorded for the G5P5 and G3P7 groups (Figures 3D–G). No significant difference in the data was observed for the G5P5 and G3P7 groups.

**FIGURE 4:**
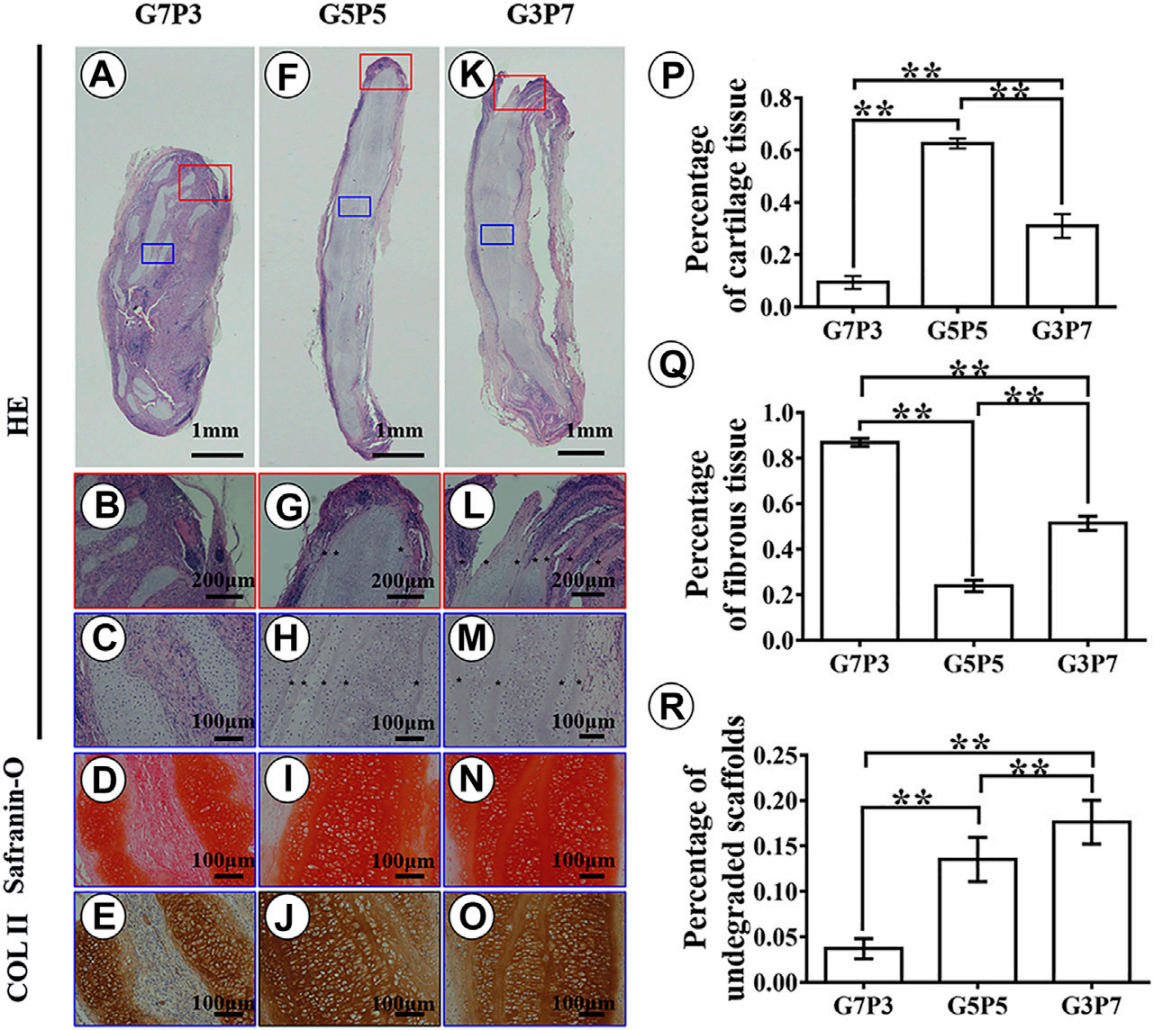
Histological examination and semiquantitative analysis in engineered tissues at 3 weeks *in vivo*. In the G7P3 group, the samples show sparse cartilage islands surrounded by abundant inflammatory cells, fibrous tissue, and discontinuous laminar scaffolds **(A–E)**. In the G5P5 and G3P7 groups, relatively homogenous cartilage is observed in the interior regions (blue box) of the samples with typical lacuna structure and positive staining of GAG and type II collagen **(H–J,M–O)**, and continuous undegraded lamellar scaffolds (marked with *) are still observed among neocartilage **(H,M)**. At the edge of the samples (red box), the G5P5 group shows relatively homogenous cartilage formation with fine interlayer integration and no obvious fibrous tissue invades toward the interior of the sample **(G)**. In the G3P7 group, relatively inferior tissue integration is observed at the edge of the samples and obvious fibrous tissue invades toward the interior of the sample along with the interlayer gap **(L)**. The semiquantitative analysis shows significant differences in area percentage of cartilage tissue, fibrous tissue, and undegraded scaffolds among the three groups **(P–R)**. *Indicating significant differences (*p < 0.05; **p < 0.01).

Results obtained by conducting histological evaluations agreed well with the results obtained from gross view and quantitative analyses. Samples belonging to the G7P3 group formed sparse cartilage islands surrounded by abundant inflammatory cells and fibrous tissues (continuous lamellar scaffolds were absent), indicating that low PCL content was not favorable for cartilage regeneration in a large animal model (Figures 4A–E). The results did not reflect the results obtained by conducting *in vitro* studies. The results also did not agree well with the results obtained by studying a nude mouse model (Figure 2) (Zheng et al., 2014). The cartilage formed in the interior regions of the samples belonging to the G5P5 and G3P7 groups were more homogenous than those formed in the interior regions of the samples belonging to the G7P3 group. These cartilage were characterized by a typical lacuna-like structure. Positive staining of GAG and type II collagen (exception: some undegraded lamellar scaffolds) was observed in the neocartilage (Figures 4F–O). The formation of homogenous cartilage with fine interlayer integration was observed at the edge of the samples belonging to the G5P5 group. Significant levels of invasion of the fibrous tissues or inflammatory cells toward the interior of the sample were not observed (Figure 4G). The extent of tissue aggregation observed at the edge of the samples belonging to the G3P7 group was relatively lower than the extent of tissue integration observed in the G5P5 group. A significant amount of the fibrous tissues invaded the interior of the sample along with the lamellar scaffolds (Figure 4L). These results indicated that a significantly high PCL content was not favorable for cartilage regeneration in a large animal model.

The results obtained from semiquantitative analyses revealed significant differences among the three groups in terms of the area percentages corresponding to the cartilage tissues, fibrous tissues, and undegraded scaffolds (Figures 4P–R). According to the statistical results, the samples belonging to the G5P5 group presented the maximum percentage for the cartilage tissue area, the minimum percentage for the fibrous tissue area, and a moderate percentage for the undegraded scaffold area. This indicated that an optimum GT to PCL ratio helped the extent of cartilage regeneration achieved. It also helped reduce the extent of invasion of the fibrous tissue in a large animal model.

### 3.5 Influence of the GT/PCL Ratio on the Maturation of the Subcutaneous Cartilage

**FIGURE 5:**
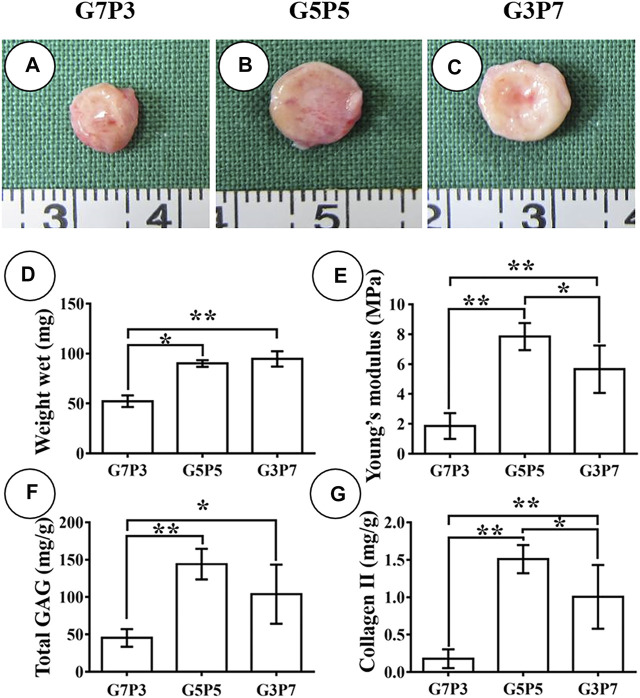
Gross view and quantitative analysis of engineered tissues at 6 weeks *in vivo*. The samples in the G7P3 group remarkably shrink **(A)** while the samples in the G5P5 and G3P7 groups retain the original size **(B,C)**. Quantitative analysis reveals that the samples in the G7P3 group show lower values in wet weight, total GAG, Collagen Ⅱ, and Young’s modulus **(D–G)** than those in the G5P5 and G3P7 groups. The samples in the G5P5 group show higher Young’s modulus and collagen II content compared to those in the G3P7 group **(E,G)**. *Indicating significant differences (*p < 0.05; **p < 0.01).

**FIGURE 6:**
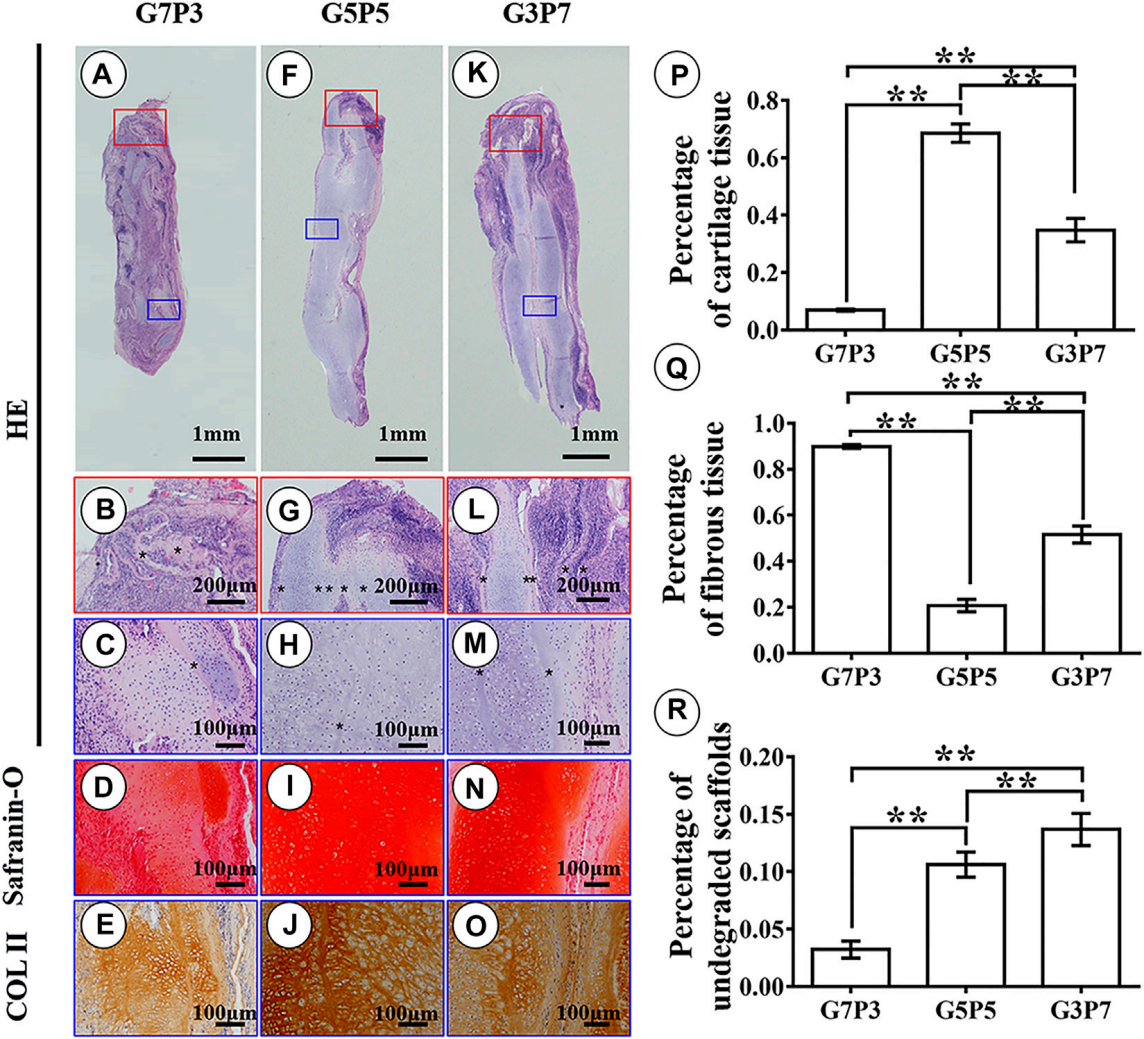
Histological examination and semiquantitative analysis in engineered tissues at 6 weeks *in vivo*. Histological evaluation shows that the samples in the G7P3 group form cartilage islands isolated by fibrous tissue and discontinuous lamellar scaffolds **(A–E)** while the samples in the G5P5 and G3P7 groups form continuous and homogenous cartilage with continuous undegraded lamellar scaffolds in the interior area of the samples **(H–J,M–O)**. The edge regions in the G5P5 group **(G)** show relatively satisfactory cartilage integration with little fibrous tissue invasion while the edge regions in the G3P7 group present relatively inferior tissue integration (separated by undegraded lamellar scaffolds *) with obvious fibrous tissue invasion **(L)**. The semiquantitative analysis shows significant differences in area percentage of cartilage tissue, fibrous tissue, and undegraded scaffolds among the three groups **(P–R)**.

We then evaluated the *in vivo* fate of the engineered cartilage after a long period (6 weeks) following subcutaneous implantation. Analysis of Figure 5 reveals that the size of the 6-week-old specimen belonging to the G7P3 group is much smaller than the size of the 6-week-old specimen belonging to the G5P5 and G3P7 groups (Figures 5A–C). The wet weight, Young’s moduli, GAG contents, and collagen II contents recorded for the samples belonging to the G7P3 groups were significantly lower than those recorded for the samples belonging to the G5P5 and G3P7 groups (Figures 5D–G). It was also observed that Young’s moduli and collagen II contents recorded for the samples belonging to the G5P5 group were higher than those recorded for the samples belonging to the G3P7 group (Figures 5E,G). The results from histological analyses further confirmed the above observation. The samples belonging to the G7P3 group formed cartilage islands isolated by fibrous tissue (Figures 6A–E), and the samples belonging to the G5P5 and G3P7 groups formed continuous and homogenous cartilage in the interior of the samples (Figures 6H–J). The extent of distribution of the cartilage and fibrous tissues realized in the samples was consistent with the distribution of the tissues realized after 3 weeks. Satisfactory levels of cartilage integration were observed in the edge regions in the samples of the G5P5 group (Figure 6G). A small extent of fibrous tissue invasion was observed in this case. Poor tissue integration and significantly high levels of fibrous tissue invasion were observed at the edges of the samples belonging to the G3P7 group (Figure 6L).

Results obtained by conducting semiquantitative analyses revealed significant differences in terms of the area percentages corresponding to cartilage tissues, fibrous tissues, and undegraded scaffolds among the three groups. The G5P5 group exhibited the best extent of cartilage formation. The formation of the minimum amount of fibrous tissues and the presence of a moderate scaffold residual were also observed in this case. This indicated that an optimum GT to PCL ratio promoted cartilage regeneration in a large animal model (Figures 6P–R). Noticeably, the area percentages corresponding to the cartilage tissues, fibrous tissues, and undegraded scaffolds recorded for the 6-week samples were consistent with the results obtained for the 3-week samples. Significant differences among the groups were not observed, indicating that the results obtained for the 3- and 6-week-old samples could predict long-term results.

### 3.6 Influence of the GT/PCL Ratio on the Inflammatory Reaction (*in vivo*)

**FIGURE 7:**
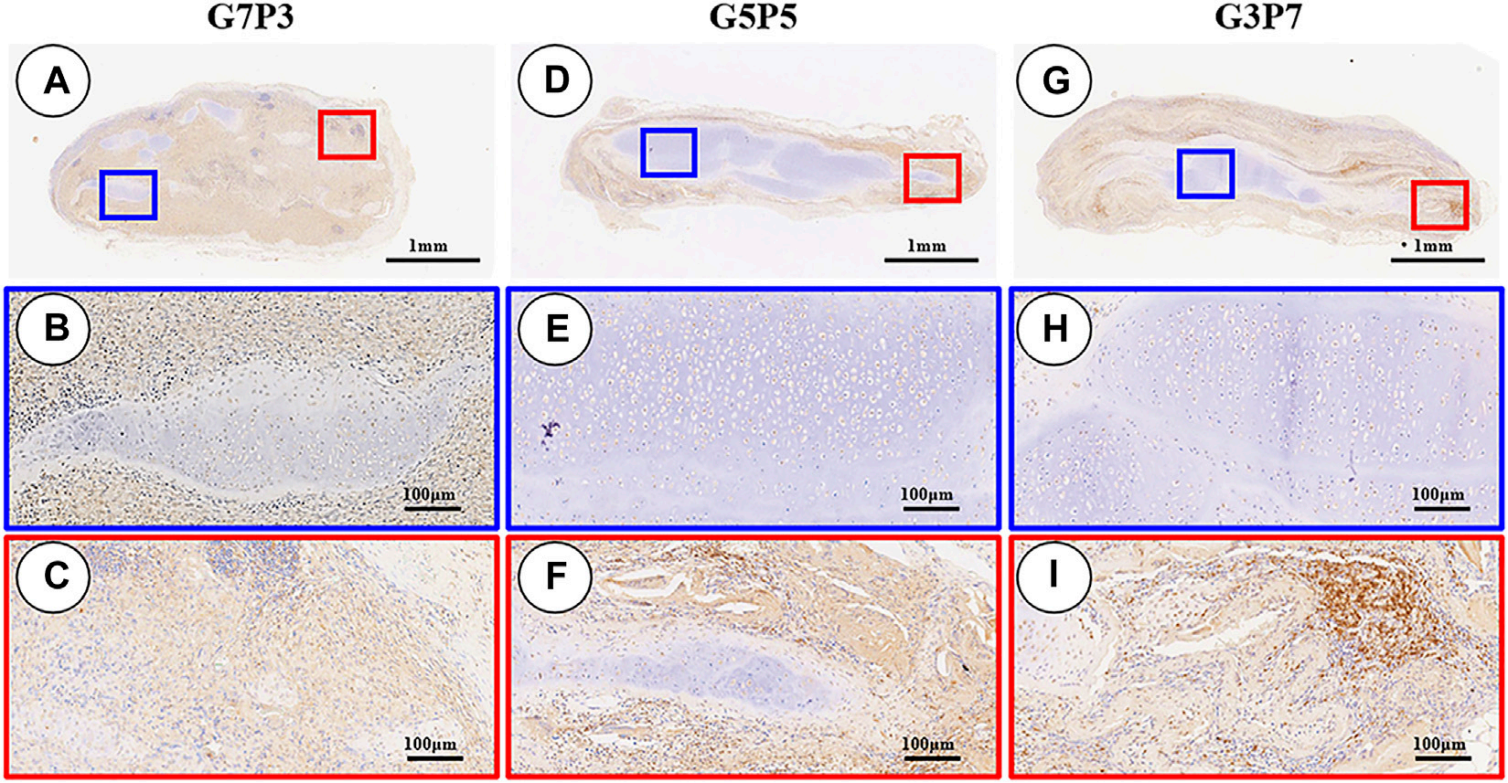
Inflammatory reaction characterized by CD3 staining. The samples in the G7P3 group show small and sparse cartilage islands surrounded by abundant CD3-positive cells **(A–C)**. In the G5P5 and G3P7 groups, CD3-positive cells are observed in the outer and edge regions but not in the central regions **(D–I)**. Compared to the G5P5 group **(F)**, more CD3-positive cells are observed among layered undegraded scaffold in the edge regions of the G3P7 group **(I)**.

**FIGURE 8:**
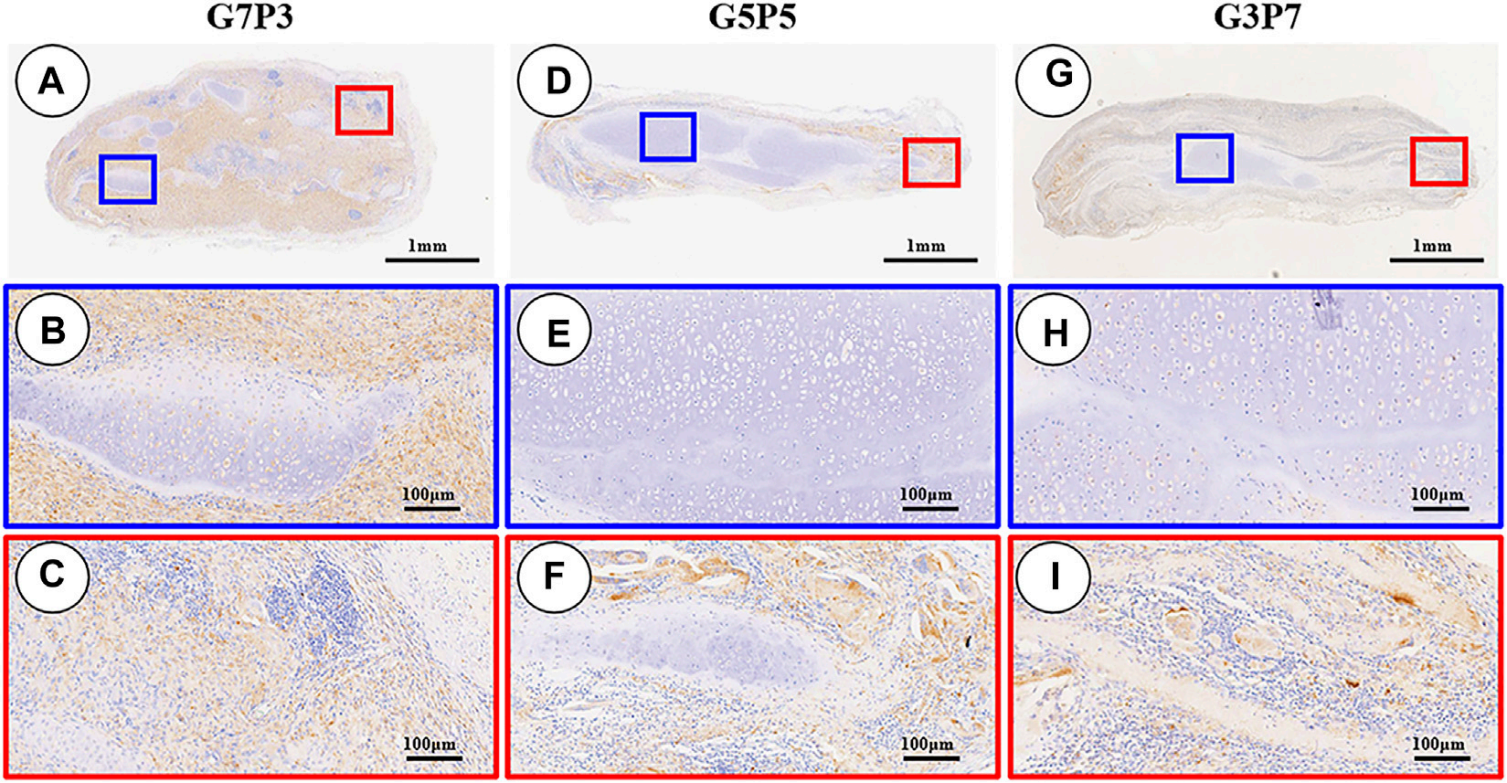
Inflammatory reaction characterized by CD68 staining. The samples in the G7P3 group show small and sparse cartilage islands surrounded by abundant CD68-positive cells **(A–C)**. In the G5P5 and G3P7 groups, CD68-positive cells are observed in the outer and edge regions but not in the central regions **(D–I)**. More CD68-positive cells are observed among layered undegraded scaffolds in the edge regions of the G3P7 group **(I)** compared to the G5P5 group **(F)**.

The 3-week specimens were further subjected to conditions of immunohistochemical staining using CD3 [T-cell marker (van Dongen et al., 1988)] and CD68 [monocyte/macrophage markers (Holness and Simmons, 1993)] to determine the status of the subcutaneous postimplantation inflammation. The results revealed that after 3 weeks of subcutaneous implantation (in autologous swine), the CD3- and CD68-positive cells presented highly consistent distribution in all the samples. The samples in different groups presented different distribution trends for CD3- and CD68-positive cells. A large number of CD3- and CD68-positive cells were observed throughout the samples surrounding the sparse cartilage islands and scaffold pieces (Figures 7A–C, Figures 8A–C) in the specimens belonging to the G7P3 group. This indicated that a low PCL content could potentially facilitate the infiltration of the inflammatory cells. In the G5P5 and G3P7 groups, CD3- and CD68-positive cells were only observed in the outer and edge regions. The presence of continuous, homogenous cartilage blocks characterized by a continuous layer of undegraded scaffolds (Figures 7D–I, Figures 8D–I) was observed at the central region. This indicated that a high PCL content could potentially help prevent the infiltration of the inflammatory cells. The presence of a large number of CD3- and CD68-positive cells was observed in the layered undegraded scaffolds at the edges of the specimens belonging to the G3P7 group. The tissue integration levels (between the layered undegraded scaffolds) (Figure 7I, Figure 8I) observed, in this case, were lower than the tissue integration levels observed in the samples belonging to the G5P5 group (with better cartilage-like tissue integration between the layered undegraded scaffolds) (Figure 7F, Figure 8F). This indicated that a significantly high PCL content might not be favorable for the prevention of inflammatory cell infiltration. This can be attributed to the low extents of cartilage regeneration and tissue integration achieved.

## 4 Discussion

The subcutaneous environment of large animals is an immune environment that is similar to the environment of the human body. Successful cartilage construction realized in the subcutaneous environment of large animals can have important guiding significance in the field of clinical transformation. We have previously reported that the electrospun GT/PCL nanofiber membrane was a promising scaffold for cartilage regeneration. We had also observed that the GT/PCL membranes characterized by low PCL content promoted cartilage construction in a nude mouse model (Xue et al., 2013; Zheng et al., 2014). The results presented herein reveal that the membranes with different GT/PCL ratios can support the process of subcutaneous cartilage regeneration in an autologous swine model. The cartilage tissues formed in the samples belonging to the G5P5 group were more homogeneous than the cartilage tissues formed in the members of the other groups. The maximum cartilage formation area ratio, the minimum extent of fibrous tissue ingrowth, and the minimum extent of infiltration of the inflammatory cells were also observed in this case. These results indicated that the GT/PCL nanofiber membrane could serve as a good scaffold that could be used to support the process of subcutaneous cartilage regeneration in future clinical translation. An optimum GT/PCL ratio helped promote cartilage formation.

The scarcity of ideal scaffolds is one of the bottlenecks that restrict the clinical translation of cartilage regeneration technology. Many scaffolds are not suitable for subcutaneous cartilage regeneration in an immunocompetent large animal model, even though these scaffolds can be used to successfully generate cartilage *in vitro* or in nude mouse models (Asawa et al., 2012; Dumont et al., 2015; Francesca et al., 2018). The unsuitability of these scaffolds can be attributed to aggressive immune activities. We have previously reported that chondrocytes combined with the PGA/PLA scaffold could be used to successfully construct cartilage *in vitro* or in nude mice. However, these could not be used to regenerate good-quality cartilage in the subcutaneous environment of large animals as severe inflammation response results in the rapid degradation of the PGA fiber (Liu et al., 2010; Luo et al., 2013). Prolonged *in vitro* pre-culture methods should be conducted (>8 weeks) to dissipate the degradation products before implantation (Liu et al., 2016; Zhou et al., 2018) to realize satisfactory cartilage regeneration. However, a long-term *in vitro* culture method is time-consuming and expensive. The process also increases the risk of contamination. The GT/PCL electrospun membrane used in the current study is composed of the naturally occurring GT and polymeric PCL. GT is an accepted biosafety scaffold that has been widely used to realize multiple tissue regeneration. However, poor mechanical strength and rapid degradation time severely restrict its application in the field of cartilage regeneration (Huang et al., 2004; Aldana and Abraham, 2017). PCL is characterized by adequate mechanical strength, high elasticity, and long degradation time and can complement the weaknesses of GT (Woodward et al., 1985; Pham et al., 2006). Importantly, PCL and GT are Food and Drug Administration (FDA)-approved non-immunogenic materials that exhibit good biocompatibility. Therefore, theoretically, the use of the GT/PCL membrane should result in the generation of less extent of immune responses in large animal models. As evidenced in the current study, the various GT/PCL membranes (differing in the GT to PCL ratios) could not easily cause the polarization of the macrophages *in vitro*. This directly proved that the materials were characterized by low immunogenicity. Following the process of subcutaneous implantation, inflammatory cell infiltrations were observed in the samples belonging to the G5P5 group, even though an abundance of the layered undegraded scaffolds was observed in the surroundings. This also indicated the low immunogenicity of the GT/PCL membranes. The chondrocyte-GT/PCL constructs cultured *in vitro* over 2 weeks formed homogenous and mature cartilage-like tissues characterized by typical lacuna-like structures. Positive staining in GAG and collagen II was observed after 3 and 6 weeks of subcutaneous implantation in the autologous swine model. This indicated that the GT/PCL membranes could serve as a promising scaffold to realize subcutaneous cartilage regeneration. The use of these membranes could significantly save the *in vitro* culture time, reduce culture costs, and reduce the risk of contamination.

The quality of the engineered cartilage presented significant differences among the three groups varying in the GT/PCL ratio in the autologous swine model. It was also observed that the influence of the GT/PCL ratio on the efficiency of cartilage regeneration in the swine model did not agree with the influence exerted on the process of cartilage regeneration in a nude mouse model (Zheng et al., 2014). In some cases, opposite trends were observed. We had previously reported that the low-PCL-ratio group could be used to realize the best cartilage regeneration in nude mice. However, the results obtained by studying the autologous swine model indicate that in the presence of the low-PCL-ratio group, the worst cartilage regeneration was realized after 3 and 6 weeks of subcutaneous implantation. A significant extent of infiltration of the inflammatory cells and high levels of ingrowth of fibrous tissues throughout the sample were observed in the samples belonging to the G7P3 group. It was speculated that the nonspecific inflammatory response caused by implantation trauma, changes in the membrane structure, and the rapid degradation of the scaffolds resulted in poor cartilage regeneration in the samples belonging to the G7P3 group. Although the GT/PCL membranes hardly triggered immune responses, the process of implantation could inevitably cause acute trauma and nonspecific inflammatory responses, resulting in the high extent of infiltration of the inflammatory cells and secretion of inflammatory factors. The rapid change in the structure of the membrane with the extent of degradation also contributed to the process. Histological analysis reveals that the membranes with different GT/PCL ratios exhibited a barrier effect that helped block the infiltration of the macrophages (*in vitro*). The rapid degradation of the scaffold, attributable to the high GT content, resulted in the weakening of the barrier effect in the samples belonging to the G7P3 group. The barrier effect observed in this case was weaker than the barrier effect observed in the other two groups. The weakening was initiated on day 6. The GT/PCL membranes subjected to the process of subcutaneous implantation failed to retain their continuous layered structures and thus failed to block the infiltration of the inflammatory cells. It was also observed that the implanted samples cultured *in vitro* over a period of 2 weeks presented loose cartilage structures. This significantly facilitated the infiltration of the inflammatory cells toward the interior of the samples. As a result, the process of cartilage regeneration in the samples of the G7P3 group was significantly disrupted by the presence of inflammatory cells and factors. These results indicated that the GT/PCL membranes characterized by high GT contents could not promote the process of subcutaneous cartilage regeneration in a large animal model.

It was also observed that a high PCL content significantly delayed the process of scaffold degradation in the samples belonging to the G5P5 and G3P7 groups. Thus, the GT/PCL membranes retained the continuous layered structures, and the cell barrier effect was also observed. Nanoscale micropores (the pore size was smaller than the cell size) were present on the continuous layered structures. The presence of these pores can potentially help block the infiltration of the inflammatory cells and macromolecule factors. They can also allow the free transportation of nutrients and metabolized products. These pores potentially impart anti-inflammatory effects to the electrospun nanofibrous membranes (Wang et al., 2016). The results presented herein confirm the feasibility of the concept. In the samples belonging to the G5P5 and G3P7 groups, the CD3- and CD68-positive cells were only observed in the outer regions and edges of the samples. These cells were absent in the inner regions, indicating that the continuous layered structures formed an anti-inflammatory barrier that helped block the invasion of the inflammatory cells. The presence of continuous, homogenous, and stable cartilage was observed in the central region, indicating that the continuous undegraded GT/PCL membranes had no significant influence on the process of transportation of nutrients and metabolized products.

The results indicated that the GT/PCL membranes characterized by high PCL contents could better retain the continuous layered structures. The membranes helped block the infiltration of the inflammatory cells. This, in turn, helped enhance the quality of the engineered cartilage. It was also observed that a significantly high PCL content might not be favorable for the prevention of the process of inflammatory cell infiltration. The number of the CD3- and CD68-positive cells present in the layered undegraded scaffold at the edges of the samples belonging to the G3P7 group was higher than the number of the CD3- and CD68-positive cells present in the samples belonging to the G5P5 group. A relatively low extent of tissue integration (compared to the extent of tissue integration realized in the G5P5 group) between the layered undegraded scaffold was observed at the edges of the samples belonging to the G3P7 group. Better cartilage-like tissue integration was realized at the edge regions of the samples belonging to the G5P5 group. The low extent of tissue integration recorded for the G3P7 group characterized by a significantly high PCL content resulted in an increase in the extent of infiltration of the inflammatory cells. Better tissue integration in the G5P5 group was achieved under conditions of optimum PCL content. The infiltration of the inflammatory cells and factors could be blocked under these conditions. The poor extent of tissue integration observed in the G3P7 group can be potentially attributed to the hydrophilicity and mechanical strength of the high PCL-content material. We have previously reported that a high PCL content in the membranes hindered the process of early cartilage formation (*in vivo*) in 3-week-old nude mice (Zheng et al., 2014). Therefore, only the GT/PCL membranes characterized by an optimum GT/PCL ratio could serve as a satisfactory scaffold for subcutaneous cartilage formation in a large animal model.

## 5 Conclusion

Homogenous cartilage could be successfully regenerated in the subcutaneous environment of an autologous swine model using electrospun GT/PCL nanofibrous membranes and autologous auricular chondrocytes following 2 weeks of *in vitro* culture. A sandwich construction strategy was followed to regenerate the cartilage. The samples characterized by an optimum GT/PCL ratio (GT: PCL = 50:50) could be used to achieve efficient cartilage regeneration. The anti-inflammatory barrier of the nanofibrous membrane and the extent of tissue integration significantly influenced the process. The samples containing a large amount of GT (GT: PCL = 70:30) could not be used for efficient cartilage formation as under these conditions, the anti-inflammatory barrier attributable to the rapid degradation of the scaffold weakened. A poor extent of tissue integration was realized under conditions of high PCL contents. Thus, the samples characterized by high PCL contents (GT: PCL = 30:70) could not be used for effective cartilage regeneration at the edges. The results presented herein provide important information and a valuable model to realize effective subcutaneous cartilage regeneration in immunocompetent animals. The results can potentially help in the clinical translation of tissue-engineered cartilage.

## Data Availability

The original contributions presented in the study are included in the article/Supplementary Material; further inquiries can be directed to the corresponding authors.
