# Supplementary material for: Regeneration of Subcutaneous Cartilage in a Swine Model Using Autologous Auricular Chondrocytes and Electrospun Nanofiber Membranes Under Conditions of Varying Gelatin/PCL Ratios
Source: Front Bioeng Biotechnol. 2021 Dec 21;9:752677. doi: 10.3389/fbioe.2021.752677 (PMC8724256; doi:10.3389/fbioe.2021.752677)
Supplement: Supplementary file 1 [file DataSheet1.PDF]

Supplementary Material

1 Supplementary Figures

1.1 Supplementary Figure 1

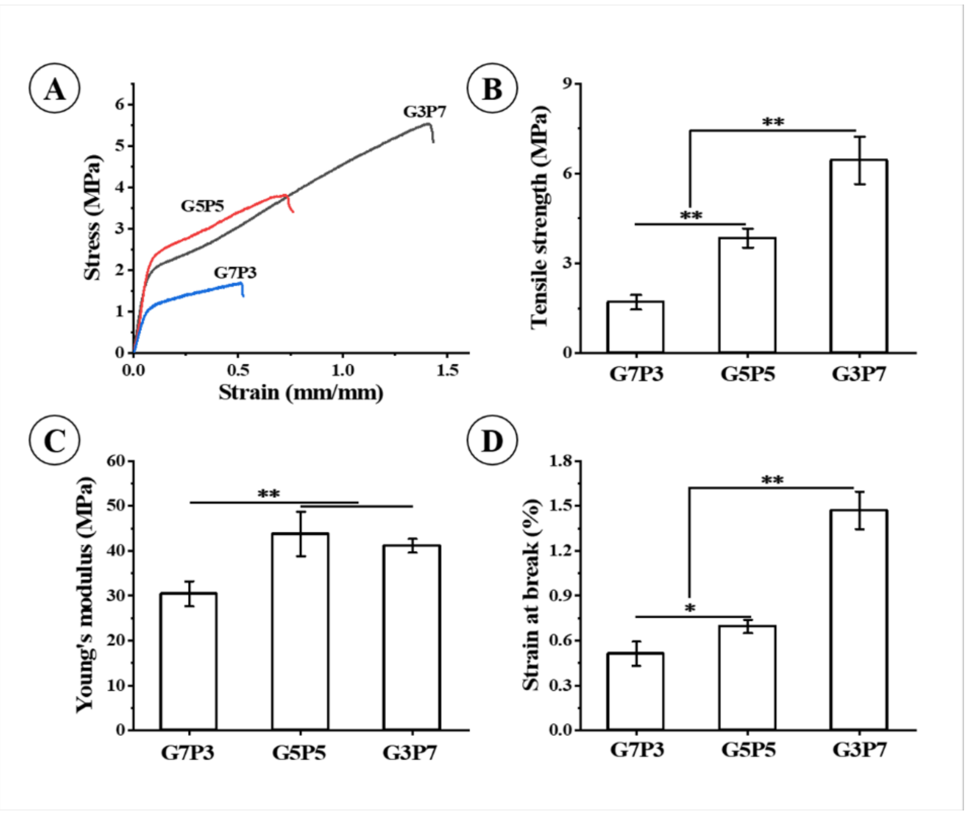

Figure S1. Mechanical analysis of GT/PCL membranes. The stress-strain curves in dry state show quite different profiles in different groups (A). Tensile strength (B) and Young's modulus (C) of the membranes increase significantly with the PCL content, while strain at break (D) shows a contrary trend. *Indicating significant differences (*p < 0.05; **p < 0.01).

1.2 Supplementary Figure 2

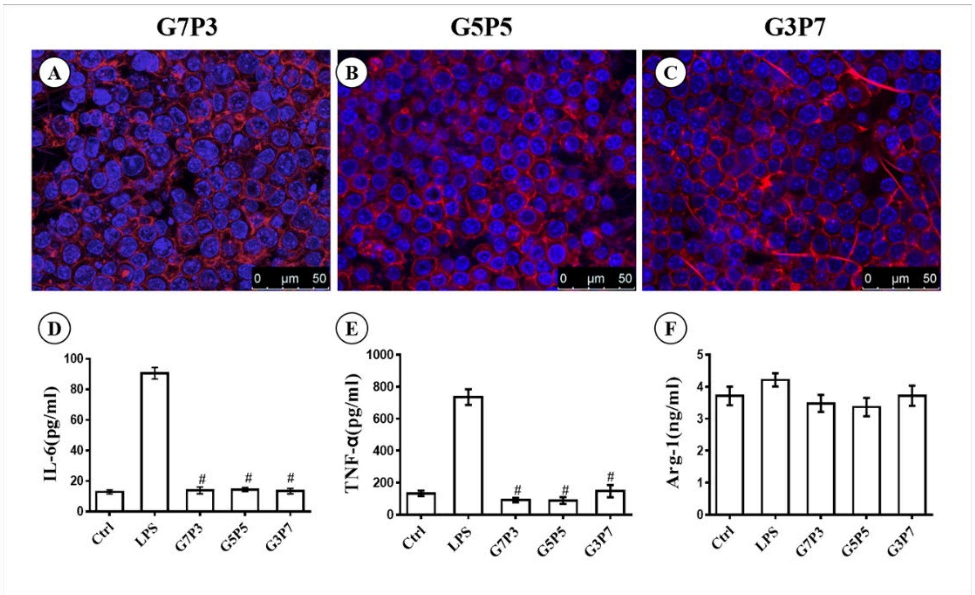

Figure S2. Biocompatibility and immunomodulatory behaviors of GT/PCL membranes. Confocal microscopy images of Raw264.7 macrophages growing on membranes with different GT/PCL content after 24 h of incubation (A-C). The blue, and red colors represent DAPI, and F-actin respectively. TNF-α (M1 marker), IL-6(M1 marker) and Arg-1 (M2 marker) cytokine secretion of Raw264.7 macrophages cultured on GT/PCL membranes after 24 h of incubation (n= 4) (D-F). *:p< 0.05versus untreated and #p< 0.05 versus LPS.

1.3 Supplementary Figure 3

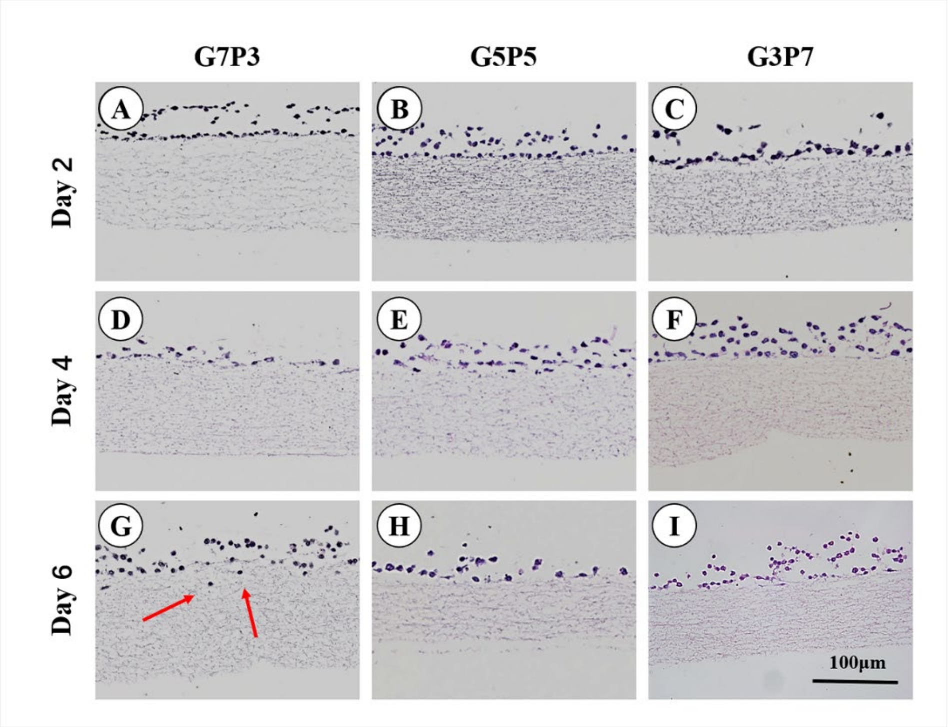

Figure S3. Macrophage distribution on GT/PCL membranes. HE staining of macrophage on GT/PCL membranes at 2, 4, 6 days(D-L). The arrows are pointing to the macrophages that have penetrated into the G7P3 membranes.
